# Alveolar Rhabdomyosarcoma of the foot metastasizing to the Iris: report of a rare case

**DOI:** 10.1186/s12885-016-2496-6

**Published:** 2016-07-11

**Authors:** Ido Didi Fabian, G. Darius Hildebrand, Shaun Wilson, Tina Foord, Mandeep S. Sagoo

**Affiliations:** Ocular Oncology Service, Moorfields Eye Hospital, 162 City Road, London, EC1V 2PD UK; St. Bartholomew’s Hospital, London, UK; Oxford Eye Hospital, The John Radcliffe Hospital, Oxford University Hospitals, Oxford, UK; Paediatric Haematology/Oncology Department, John Radcliffe Hospital, Oxford University Hospitals, Oxford, UK; Churchill Hospital, Oxford University Hospitals, Oxford, UK; UCL Institute of Ophthalmology, London, UK

**Keywords:** Rhabdomyosarcoma, Iris, Metastasis, Case report

## Abstract

**Background:**

Intraocular iris rhabdomyosarcoma is extremely rare, and in the 3 cases reported to date occurred as the primary site of tumour growth. We report a case of rhabdomyosarcoma of the foot metastasizing to the iris.

**Case presentation:**

An 18-year-old white female was referred to the London Ocular Oncology Service for management of a metastatic rhabdomyosarcomatous deposit in the iris, a metastasis from alveolar rhabdomyosarcoma of the foot. She was diagnosed nearly 2 years earlier with the primary sarcoma with extensive systemic spread and treated by resection of the foot lesion and chemotherapy, and achieved a partial remission. The left iris deposit was noted while she was receiving systemic chemotherapy, heralding a relapse. However, anterior uveitis and raised intraocular pressure developed and she was referred to our service for further management. A left iris secondary rhabdomyosarcoma deposit was noticed and in addition a lacrimal gland mass, as indicated by ultrasound B scan of the eye and orbit. The patient was treated with external beam radiotherapy to the globe and orbit, but died 2 months after treatment completion.

**Conclusion:**

Rhabdomyosarcoma of the iris is very rare and was previously documented only as a primary malignancy in this location. We report that secondary spread to the iris can also occur, in this case as the first sign of widely disseminated systemic relapse.

## Background

Rhabdomyosarcoma (RMS) is the most common soft tissue sarcoma in the paediatric population [1]. The orbit is the primary tumour site in 10 % of cases and is rarely a site for secondary spread from a distant extra-orbital origin [2]. Intraocular primary RMS of the uvea is yet another rare presentation of the disease, described only in a handful of case reports [2]. Herein, we report a unique case of secondary RMS to the iris, a metastasis from alveolar RMS of the foot.

## Case presentation

An 18-year-old white female was referred to the London Ocular Oncology Service for management of a metastatic RMS deposit of the left iris. She was originally diagnosed by the Oxford Oncology team with metastatic alveolar RMS 22 months earlier, with the primary in the right flexor digitorum brevis, with extensive metastatic disease, including intramuscular deposit to the calf, popliteal fossa nodes, external inguinal chain, multiple mediastinal nodes and bilateral lung parenchymal involvement with pleural effusion. Systemic chemotherapy (ifosfamide, vincristine, actinomycin and doxorubicin) with surgical resection of the right foot lesion, followed by maintenance chemotherapy achieved prolonged disease control.

**Fig. 1:**
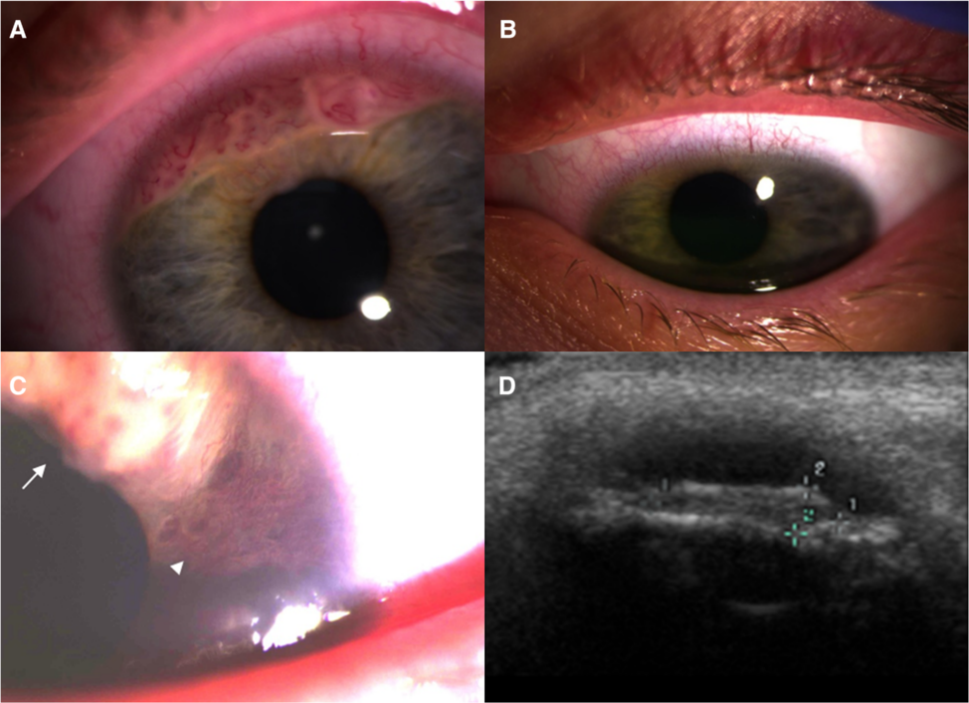
Left eye iris mass at presentation (**a**), resolved after initial systemic chemotherapy (**b**). Ocular tumour relapse (*arrow*) and neovascularization of the iris (*arrowhead*) (**c**), demonstrated also on B mode ultrasound scan (**d**)

Fourteen months after diagnosis of the metastatic RMS she noticed a change in the left iris colour. Examination done at the Oxford Eye Hospital indicated an iris mass (Fig. 1a), and on PET scan, extensive systemic relapse was detected, including uptake of the primary foot site, pulmonary nodules as well as the left iris. Vincristine, irinotecan and temozolomide (VIT) were started, to control the recurrent disease, including the iris metastasis, which showed good response (Fig. 1b). A subsequent on – treatment relapse occurred after 6 cycles of VIT (primary site, lungs and musculoskeletal). Palliative oral etoposide was commenced 5 months after first intraocular involvement, keeping the disease under relative control.

After 3 months of palliative oral etoposide, the iris tumour recurred, with neovascularization (Fig. 1c), anterior uveitis and intraocular pressure of 40 mmHg. She was treated with topical antihypertensives and steroids and referred to our service for consideration for radiotherapy.

Visual acuity was 6/6 in the right eye and 6/36 in the left eye. Intraocular pressures were 14 mmHg RE and 15 mmHg LE. The right eye was entirely normal. The left eye had a ciliary flush. There were keratic precipitates but also larger areas of more confluent deposits on the corneal endothelium, with stromal oedema. An amelanotic multifocal vascularised mass was present in the superior and inferior aspect of the iris, occluding most of the iridocorneal angle. There were no ciliary body or choroidal tumours, but cupping of the optic disc was noted. B mode ultrasound scan (Acuson Sequoia 512, Siemens AG, Munich, Germany) with a 14 MHz linear B-scan array probe indicated a 1.7 mm elevated iris lesion with irregular anterior borders (Fig. 1d) and no ciliary body involvement, and in addition a lacrimal gland mass was noted. The patient was treated with palliative external beam radiotherapy (20Gy in 5 fractions) for both presumed intraocular and orbital metastatic RMS deposits. Treatment was well tolerated and there was a prompt and sustained clinical benefit. She died 2 months later (2 years after RMS diagnosis) from systemic progressive disease.

## Discussion

There is a wide range of tumours that occur in the iris. Metastasis to the iris is infrequent, reported in fewer than 10 % of intraocular secondary deposits by Shields et al. and Konstantinidis et al. [3, 4]. In both reports the most common primary site was breast and lung, and there were no cases of systemic RMS metastasizing to the eye; hence the present case is highly unusual.

In another series by Shields and colleagues, the clinical features of 104 patients with iris metastasis from systemic cancer were reported [5]. The median age was 60 years, most were white females, the main symptoms were pain or blurred vision and the main findings were corectopia and secondary glaucoma. Most tumours were unifocal and found in the inferior quadrant. In the present case, the patient was 18 years old when the intraocular mass was detected; she had no ocular symptoms but incidentally noticed iris heterochromia; the initial iris relapse was in the superior quadrant and only further relapse occurred superiorly and inferiorly, causing secondary glaucoma. In agreement with other reports, metastasis to the iris is a feature of advanced disseminated cancer with poor life prognosis [5].

RMS of the iris has been previously described, but is considered to be an extremely rare occurrence, reported to date only in 3 patients [6–8]. In all of these cases, the iris was the primary site of tumour growth. The Table summarizes the main clinical features found in those cases and in the present one. The age at presentation of patients with primary iris RMS was 5 years or younger and the presenting feature was development of an iris mass. Definitive treatment in those cases ultimately was enucleation with no local or systemic sequelae. The present case differed from the primary iris RMS ones in nearly all clinical features, but the mode of presentation. Management and outcome were obviously different.

RMS is classified into 4 histopathological types: embryonal, alveolar, botryoid and pleomorphic [2]. Orbital alveolar type is considered less common but more aggressive than the embryonal type [9]. The relation between the cell type, incidence and prognosis of intraocular RMS is not well established, since this is a rare occurrence.

## Conclusion

Rhabdomyosarcoma of the iris is rare. Not only can this tumour develop in the iris as a primary site [10], but we report that secondary spread to the iris can also occur, in this case as the first sign of widely disseminated systemic relapse.

## Abbreviations

RMS, rhabdomyosarcoma

## References

[1] Crist W, Gehan EA, Ragab AH, Dickman PS, Donaldson SS, Fryer C, Hammond D, Hays DM, Herrmann J, Heyn R (1995). The third intergroup Rhabdomyosarcoma study. J Clin Oncol.

[2] Shields JA, Shields CL (2003). Rhabdomyosarcoma: review for the ophthalmologist. Surv Ophthalmol.

[3] Shields CL, Shields JA, Gross NE, Schwartz GP, Lally SE (1997). Survey of 520 eyes with uveal metastases. Ophthalmology.

[4] Konstantinidis L, Rospond-Kubiak I, Zeolite I, Heimann H, Groenewald C, Coupland SE, Damato B (2014). Management of patients with uveal metastases at the Liverpool Ocular Oncology Centre. Br J Ophthalmol.

[5] Shields CL, Kaliki S, Crabtree GS, Peshtani A, Morton S, Anand RA, Coco G, Shields JA (2015). Iris metastasis from systemic cancer in 104 patients: the 2014 Jerry A. Shields Lecture. Cornea.

[6] Woyke S, Chwirot R (1972). Rhabdomyosarcoma of the iris. Report of the first recorded case. Br J Ophthalmol.

[7] Font RL, Zimmerman LE (1972). Electron microscopic verification of primary rhabdomyosarcoma of the iris. Am J Ophthalmol.

[8] Elsas FJ, Mroczek EC, Kelly DR, Specht CS (1991). Primary rhabdomyosarcoma of the iris. Arch Ophthalmol (Chicago, Ill 1960).

[9] Kodet R, Newton WA, Hamoudi AB, Asmar L, Wharam MD, Maurer HM (1997). Orbital rhabdomyosarcomas and related tumors in childhood: relationship of morphology to prognosis--an Intergroup Rhabdomyosarcoma study. Med Pediatr Oncol.

[10] Shields CL, Shields JA, Honavar SG, Demirci H (2001). Clinical spectrum of primary ophthalmic rhabdomyosarcoma. Ophthalmology.

